# Generalizing multiple memories from a single drive: The hysteron latch

**DOI:** 10.1126/sciadv.adr5933

**Published:** 2025-01-29

**Authors:** Chloe W. Lindeman, Travis R. Jalowiec, Nathan C. Keim

**Affiliations:** ^1^ James Franck Institute and Department of Physics, University of Chicago, Chicago IL, USA.; ^2^ Department of Physics, Pennsylvania State University, University Park, PA, USA.

## Abstract

Far-from-equilibrium systems can form memories of previous deformations or driving. In systems from sheared glassy materials to buckling beams to crumpled sheets, this behavior is dominated by return-point memory, in which revisiting a past extremum of driving restores the system to a previous state. Cyclic driving with both positive and negative strains forms multiple nested memories, as in a single-dial combination lock, while asymmetric driving (only positive strain) cannot. We study this case in a general model of hysteresis that considers discrete elements called hysterons. We show how two hysterons with a frustrated interaction can violate return-point memory, realizing multiple memories of asymmetric driving. This reveals a general principle for designing systems that store sequences of cyclic driving, whether symmetric or asymmetric. In disordered systems, asymmetric driving is a sensitive tool for the direct measurement of frustration.

## Teaser

Interacting elements with hysteresis store a sequence of amplitudes in a unique way, functioning as a combination lock.

## INTRODUCTION

The single-dial combination lock is a mechanism for storing multiple values from a single input. By alternating between clockwise and counterclockwise rotation, the operator encodes the combination values as a series of turning points. Each new turning point must be nested within the previous two, so that the lock verifies not only the values but also their exact sequence. Information about the stored values can be recovered by observing the resistance to further rotation (*1*). Last, erasure can be achieved with a large twist of the dial. Every operation is accomplished with a single control.

**Fig. 1.**
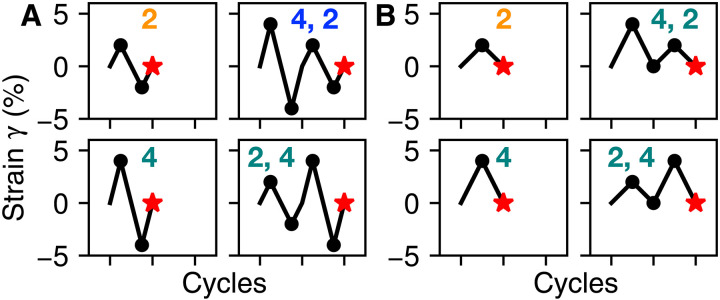
Driving protocols can store multiple memories. The state of a generic system with return-point memory depends on the sequence of nested turning points from driving. (**A**) Driving protocols that store the amplitude(s) of symmetric (positive and negative) shear; dots mark turning points. Final memory-encoding state corresponds to red stars. “2,” “4,” and “4, 2” each lead the system to a different state. “2, 4” does not nest turning points within preceding ones, and so yields the same state as “4.” (**B**) Asymmetric equivalents of (A). Because every cycle has the same turning point at 0, it is impossible to store multiple values via return-point memory.

This elegant idea was known by 1909 (*2*), but it was rediscovered decades later as return-point memory, a generic behavior of many materials and systems that contain hysteretic elements, even those that appear quite different from the series of wheels in a combination lock (*1*, *3*–*5*). For example, an amorphous solid that is repeatedly sheared back and forth contains many localized groups of rearranging particles (*6*–*8*). In experiments, forward and reverse shear drive each of these groups to switch its configuration reversibly between a “forward” (“+”) and “reverse” (“−”) state, but only when a sufficiently large deformation is applied in each direction, meaning that the rearrangements have hysteresis. Collectively, the states of these rearrangements uniquely encode the nested turning points of shear strain (illustrated in Fig. 1A), and a suitable readout protocol can recover some or all of that history (*6*, *9*). Unlike in the combination lock, the memory is explained by the observation that rearranging groups act as “hysterons,” bistable elements that are the building blocks of many models of hysteresis (*10*). The way return-point memory arises in ensembles of independent hysterons is rigorously understood (*4*, *11*), and the same picture emerges from observations of creases in crumpled sheets (*12*) and buckling units in mechanical metamaterials (*13*, *14*); from models of rocks (*15*) and ferromagnetic, ferroelectric, and martensitic materials (*1*); and from many more systems (*5*, *16*, *17*).

In some of these systems, interactions between hysterons are also apparent. In the case of the amorphous solid, the minimum imposed strain needed to drive a group of rearranging particles into the + state might be raised or lowered according to the state of another nearby group, due to their coupling via the material between them. Of particular interest are “frustrated” or antiferromagnetic interactions that make it harder to drive a pair of hysterons into the same state. When present among large numbers of hysterons or other elements that relax, frustrated interactions are associated with the extraordinarily rugged energy landscapes of glassy materials like amorphous solids and crumpled sheets (*12*, *18*). Recent simulations of hysterons with frustrated interactions have explored intriguing behaviors that are inconsistent with return-point memory, such as counting cycles of driving (*19*–*22*), and interacting mechanical hysterons have now been demonstrated in experiments (*5*, *13*, *14*, *23*, *24*).

Theoretically, the behaviors of interacting hysterons can be arbitrarily complex computations, given enough hysterons and the precise control of their parameters (*23*, *25*). Yet the example of return-point memory shows that a useful and capacious memory behavior, the ability to store sequences of turning points, can instead arise from an ensemble of simple units (hysterons), making it ubiquitous in both designed and disordered systems. Encoding longer sequences merely involves expanding the ensemble size, much as one would add more wheels to the mechanism of a combination lock. This example leads us to ask whether, among the plethora of behaviors enabled by hysterons’ interactions, there is an analogous form of memory based on repeating a minimal unit.

Here, we show that return-point memory has a direct analog in hysterons with frustrated interactions. While return-point memory stores the turning points of symmetric driving (Fig. 1A), frustrated interactions let hysterons store the turning points of asymmetric or rectified driving (Fig. 1B), such as when a bridge is crossed by a series of vehicles with different weights. In contrast, any system with pure return-point memory, including the combination lock, can store only the largest turning point of asymmetric driving.

Just as the hysteron is the repeated unit that gives rise to return-point memories, we find that the unit for nested memories of asymmetric driving is nearly as elemental: just two hysterons with a frustrated interaction. This pairwise mechanism was recently confirmed experimentally by Paulsen (*24*), with hysterons realized as two bistable rotors coupled via springs to each other and to a driving rod. From this basic unit of memory, we formulate a general principle of “latching”: conditions for preventing the system from returning to its previous state, which allow multiple memories to be stored with either symmetric or asymmetric driving. Our results point the way to targeted studies of interacting relaxations in disordered matter and to the rational design of information-processing capabilities in mechanical systems.

### Model

We are motivated by models of disordered solid materials and metamaterials, wherein a system contains mesoscopic hysterons that can reversibly switch between two states under an imposed strain γ, for example, by rearrangements of particles in an amorphous solid (*7*, *26*, *27*) or snap-through of a buckled beam (*13*, *28*). These models capture many mesoscopic details of experiments and simulations (*6*, *7*, *13*, *14*, *22*, *23*, *29*), especially when hysterons can interact. The *i*th hysteron switches its state $S_{i}$ at thresholds $\gamma_{i}^{\pm }$: When $\gamma >\gamma_{i}^{+}$, $S_{i}=+1$; when $\gamma <\gamma_{i}^{-}$, $S_{i}=-1$; and when $\gamma_{i}^{-}<\gamma <\gamma_{i}^{+}$, $S_{i}$ remains in its previous state. For convenience, we write states as + and −. In our simulations, the “system” is an ensemble of many groups of *N* hysterons with random parameters, modeling interactions via perturbed thresholds

$$\gamma_{i}^{\pm }\left(S_{j\neq i}\right)=\gamma_{i}^{\pm }-\sum_{j\neq i}J_{\mathrm{ij}}S_{j}$$ (1)

where $S_{j\neq i}$ represents the states of all hysterons excluding hysteron *i*, and $J_{\mathrm{ij}}$ is the N×N interaction matrix. This is equivalent to the model in (*19*). $J_{\mathrm{ij}}<0$ represents a frustrated interaction in the sense that one hysteron flip inhibits another hysteron from flipping in the same direction. Large, disordered systems with these interactions tend to be “glassy,” in that they have rugged energy landscapes with many metastable states that are far from a global energy minimum (*18*). We initially omit positive (cooperative) interactions that cause one hysteron’s flip to encourage like flips and that can lead to avalanches, because the memory behavior with those interactions is known to be qualitatively the same as with noninteracting hysterons (*4*). Accordingly, we choose $J_{\mathrm{ij}}$ with uniform probability from $\left[-J_{0},0\right]$, with $J_{0}=0.01$ unless otherwise specified. Each hysteron’s $\gamma_{i}^{\pm }$ are chosen by drawing two values from a uniform distribution on [−0.1, 0.1] and then ordering them so that $\gamma_{i}^{-}<\gamma_{i}^{+}$, corresponding to rearrangements that dissipate energy.

To simulate this model as γ is varied, we use the open-source “hysteron” software package (*19*). Like molecular dynamics simulations of amorphous solids, we work in the athermal and quasistatic limit: The algorithm identifies the hysteron that will flip soonest as γ changes and then holds γ fixed while it updates any other hysterons that were destabilized via interactions (e.g., an avalanche), starting with the hysteron farthest past its threshold. When *N* > 2, the random parameters would occasionally prevent the algorithm from finding a stable state; these cases were discarded (*19*). Unless specifically noted, we avoid the case where $J_{\mathrm{ij}}$ and $J_{\mathrm{ji}}$ have opposite signs, for which this issue is common.

## RESULTS

### Memories in noninteracting and interacting systems

**Fig. 2.**
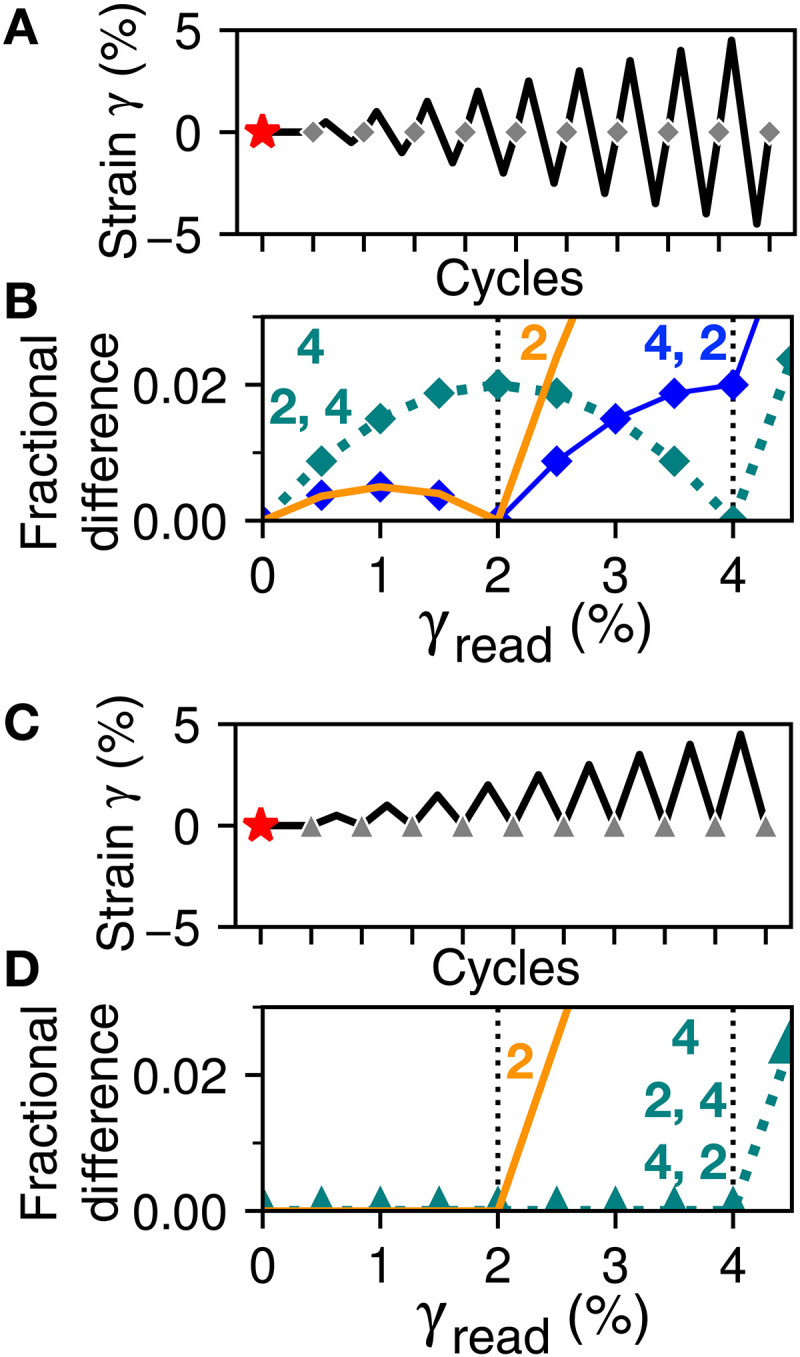
Readout confirms the turning-point analysis of Fig. 1. (**A**) Symmetric strain protocol for reading out memories. State $S_{\text{mem}}$ after writing, marked with red stars, is compared with state after each cycle (gray diamonds), as amplitude $\gamma_{\text{read}}$ increases. (**B**) Results of readout, measured as fraction of hysterons that differ. Each curve is labeled with the write protocol(s) from Fig. 1A that produced it. The most recent amplitude is always present, and multiple memories are possible if written in descending order. (**C**) Equivalent asymmetric protocol. Ends of readout cycles are marked with gray triangles. (**D**) Readout results for each protocol in Fig. 1B. The system cannot store a sequence of asymmetric driving, and the most recent amplitude may be missing.

Protocols for writing memories are shown in Fig. 1; example protocols for readout are in Fig. 2 (A and C). We begin each simulation with all hysterons in the − state, as though γ → −∞, and then we drive the hysterons to γ = 0 before encoding memories. (Starting at γ → ∞ similarly supports our conclusions; it is considered in fig. S1.)

Without loss of generality, in single-memory tests, we take the stored strain amplitude to be 4%; in two-memory tests, we use 4 and 2%; and in tests with asymmetric driving, we take the bottom turning point to be 0. The amplitudes correspond to commonly used strains in studies of memory in amorphous solids (*6*, *9*, *30*, *31*), but they can be rescaled arbitrarily to match other physical systems (Supplementary Text). After the writing cycle(s), the state is saved as $S_{\text{mem}}$. To read out the memories encoded in $S_{\text{mem}}$, we apply a series of cycles with increasing amplitude $\gamma_{\text{read}}=0,0.005,0.01\ldots$. This is a “serial” protocol that is suited to experiments, as opposed to a “parallel” protocol in which a separate copy of the system is made for each readout cycle (*31*). After each cycle with amplitude $\gamma_{\text{read}}$, we record the fraction of hysterons that are different from $S_{\text{mem}}$. We average this fraction over the entire ensemble and plot it against $\gamma_{\text{read}}$.

**Fig. 3.**
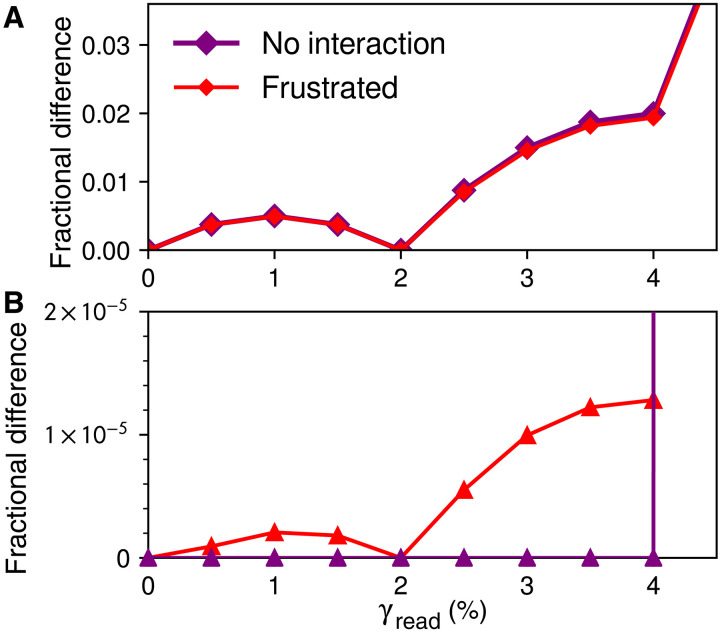
Frustrated hysteron pairs store multiple amplitudes of both symmetric and asymmetric driving. (**A**) Curves from symmetric driving have cusps at 2 and 4%. (**B**) Asymmetric driving. Without interactions, only the memory at 4% is present. Curve from 10^8^ frustrated pairs shows both memories.

Figure 3A shows readouts after the symmetric two-amplitude protocol labeled “4, 2” in Fig. 1A. The curve from an ensemble of noninteracting hysterons ($J_{\mathrm{ij}}=0$) is reproduced from the “4, 2” curve of Fig. 2B; it features a local minimum that indicates the most recent memory (2%) and a cusp that indicates the larger memory (4%), just as in the corresponding amorphous solid experiments and molecular dynamics simulations (*6*, *9*, *30*, *31*). However, in Fig. 3B, driving the same ensemble asymmetrically changes its behavior markedly: The curve shows no signature of the smaller driving amplitude, rising rapidly only after 4%.

These notable different behaviors are both expected from return-point memory, wherein the system remembers the turning points of driving. As long as γ is bounded between any pair of turning points, visiting either turning point will return the system to the state it had when it was at that turning point before (*3*, *4*). This property is recursive, meaning that one may encode more than one memory with a symmetric driving protocol by decreasing the strain amplitude, such that each new pair of turning points is nested within the last as in the “4, 2” protocol of Fig. 2A.

However, return-point memory also means that two asymmetric cycles can write only one memory: The first cycle of the “4, 2” protocol in Fig. 1B establishes a bounding turning point at γ = 0, and visiting γ = 0 again after writing the second, smaller amplitude immediately restores the state with just one memory. Repeating the results of Fig. 2D, the readout of noninteracting hysterons in Fig. 3B fails to change their states until γ = 4% is exceeded: Writing a second, smaller memory has no effect.

We now consider frustrated interactions, $J_{\mathrm{ij}}<0$, as found in models of glassy matter such as crumpled sheets, disordered or amorphous solids, or spin ice and spin glass. Frustration means that one relaxation inhibits others, leading to these materials’ characteristically rugged landscapes of metastable states with broad distributions of energy barriers (*18*). In hysteron models, the sequences in which hysterons switch during forward or reverse shear become mutable, so that return-point memory is no longer assured (*4*, *21*, *22*, *32*). The red curves of Fig. 3A show that, nonetheless, replacing the ensemble of single hysterons with an ensemble of frustrated pairs merely perturbs the return-point memory of symmetric driving. However, in Fig. 3B, where the readout curve for asymmetric driving had been zero, there is now a clear signature of both memories. This signal resembles the much larger one from symmetric driving, suggesting a connection.

### Memory mechanisms

**Fig. 4.**
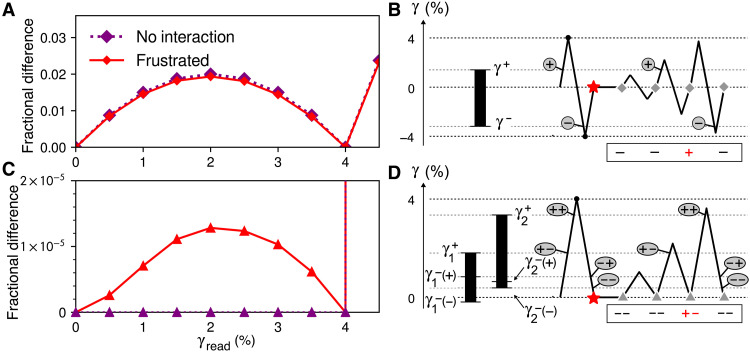
Mechanisms for memory. (**A**) Readouts of single memory of symmetric driving. Local minima indicate the memory. (**B**) Schematic of how a single hysteron contributes to memory, showing hysteron thresholds on the left and an abridged driving protocol on the right. Changes in state during readout are marked with circled + and −. State at the end of each cycle is shown below; only the + state contributes to readout. (**C**) Readouts of single memory with asymmetric driving. The curve without interactions is zero for $\gamma_{\text{read}}\leq 4\%$ and then increases, while the curve with frustration is non-monotonic. (**D**) Schematic for a frustrated pair with asymmetric driving. For clarity, we show only how the interaction splits the hysteron bottoms $\gamma_{1}^{-}$ and $\gamma_{2}^{-}$ as a function of the other hysteron’s state, because the ordering of these four values is crucial for nonzero readout. The hysteron tops $\gamma_{1}^{+}$ and $\gamma_{2}^{+}$ can vary widely (Supplementary Text).

To understand this connection mechanistically, we first return to noninteracting hysterons and examine their memory of a single amplitude, encoded with the symmetric protocol labeled “4” in Fig. 1A. Just as in the two-memory case, the corresponding readout curve in Fig. 4A is consistent with return-point memory: Each readout cycle with $\gamma_{\text{read}}<4\%$ establishes new turning points nested within the original pair at ±4%, placing the system in a new and distinct state and yielding a nonzero difference signal; when $\gamma_{\text{read}}=4\%$, the original turning points are revisited and the state at the cycle’s end matches $S_{\text{mem}}$, making the signal zero. This leads to the distinctive rise and fall of the readout curve below the training strain.

In this case, we can understand the behavior of the ensemble by studying how a single hysteron contributes to memory. Figure 4B shows the response of a particular hysteron to an abridged writing and readout protocol. Only hysterons such as this one, with $-\gamma^{-}>\gamma^{+}>0$, contribute to readout for $\gamma_{\text{read}}\leq 4\%$ because only they will be in the − state after writing, will then become trapped in the + state when amplitude is reduced, and will lastly return to − when the original amplitude is resumed. In this sense, the hysteron “latches” into the + state during intermediate strain cycles. From the broad distributions of γ^±^, this mechanism yields the smooth rising and falling curve for $\gamma_{\text{read}}\leq 4\%$ in Fig. 4A. The hysterons with other arrangements of γ^±^ end each readout cycle in the same state for $0\leq \gamma_{\text{read}}\leq 4\%$ and, hence, do not contribute to the signal below the training strain.

Just as with two memories, in Fig. 4C, frustration enables a single memory of asymmetric driving that resembles its symmetric counterpart. We show in Fig. 4D that this happens by an analogous mechanism involving a frustrated pair of hysterons. Frustration allows a two-hysteron latching behavior in which revisiting the turning point at γ = 0 can fail to restore the previous state. The mechanisms of Fig. 4 (B and C) are thus equivalent when one treats each whole cycle as one transition, either to the same state or to a new state (*33*).

### Scaling the two-hysteron latch

**Fig. 5.**
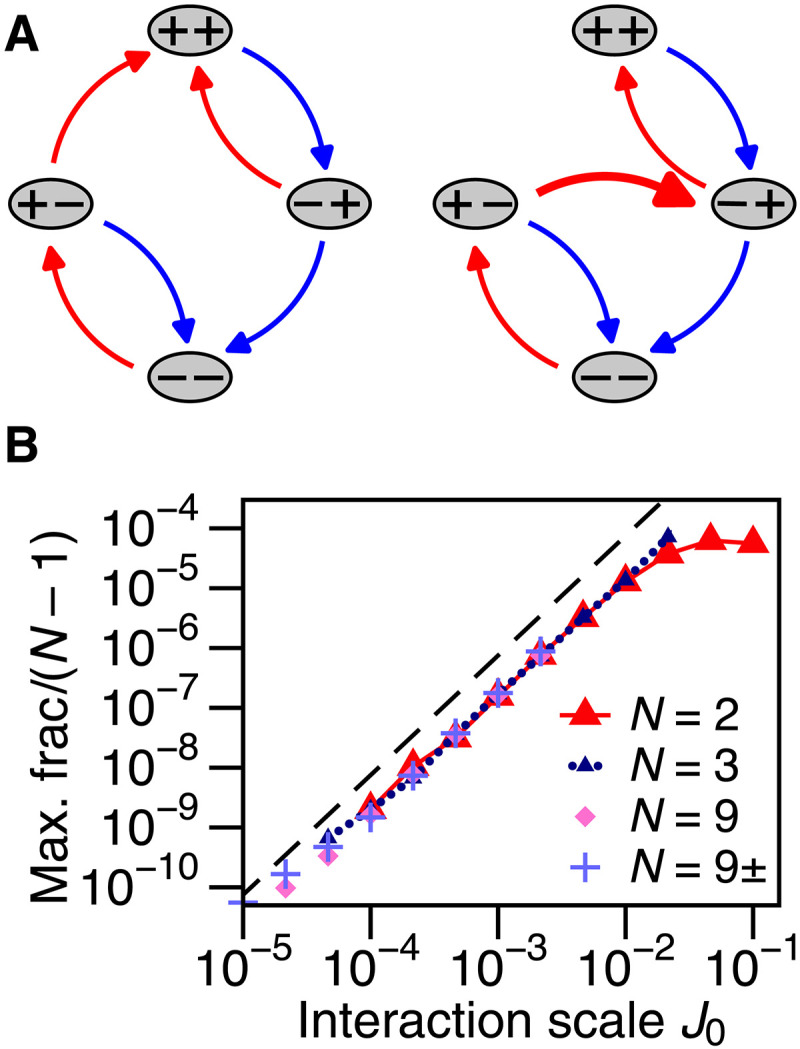
Latching pair transition graphs and scaling. (**A**) Transition graphs that can give rise to nonzero readout below the remembered strain in simulations of frustrated hysterons. Transitions while increasing strain are in red, and while decreasing strain are in blue. Left graph corresponds to Fig. 4D. Thick arrow represents an avalanche in which + − transitions to − + upon increasing strain, by way of the unstable intermediate state + +. (**B**) Peak signal in Fig. 4C for $\gamma_{\text{read}}<4\%$, varying interaction scale $J_{0}$ and number of mutually interacting hysterons *N*, scaled by the number of pair interactions per hysteron (*N* − 1). In “9±,” half of the $J_{\mathrm{ij}},J_{\mathrm{ji}}$ pairs are positive (cooperative), so data are scaled by (N−1)/2. Each point is the average of 10^9^*N* hysterons. Dashed line shows $J_{0}^{2}$ scaling for reference.

What features are needed for latching? The values $\gamma_{1}^{+}$ and $\gamma_{2}^{+}$ set the hysterons’ sensitivity to amplitude; as long as they exceed most lower thresholds, they can vary widely, creating the smooth, slightly asymmetric curve in Fig. 4C. By contrast, the lower thresholds must satisfy

$$\gamma_{1}^{-}\left(-\right)<0<\gamma_{2}^{-}\left(-\right)<\gamma_{2}^{-}\left(+\right)<\gamma_{1}^{-}\left(+\right)$$ (2)

as in Fig. 4D. These inequalities were verified in simulations by testing 10^9^ random pairs. In addition to the explicit flipping thresholds shown, it can be useful to visualize the order in which states are visited via a “transition graph.” The graphs that can lead to latching in our frustrated simulations and, hence, to nonzero readout are shown in Fig. 5A. If we let $J_{\mathrm{ij}}$ and $J_{\mathrm{ji}}$ have opposite signs, then a third transition graph can contribute (see fig. S3 for details).

The interaction strength sets the threshold “window” size $\gamma_{1}^{-}\left(+\right)-\gamma_{1}^{-}\left(-\right)=-2J_{12}$, into which both 0 and the interval $\left[\gamma_{2}^{-}\left(-\right),\gamma_{2}^{-}\left(+\right)\right]$ must fall. For ensembles with uniformly distributed parameters like those reported here, these two requirements make the probability *P* for Eq. 2 second order in the interaction strength, i.e., $P∼J_{0}^{2}$. The signals in Fig. 4C are, thus, small compared to the result from return-point memory under symmetric driving, which is zeroth order in the sense that it may be obtained with $J_{0}=0$. The $J_{0}^{2}$ scaling is confirmed for small interaction strength ($J_{0}\ll 4\%$) in Fig. 5B, where we measure the maximum height of the readout signal for $\gamma_{\text{read}}<4\%$ (see Supplementary Text for details). In a design context, our analysis means that greatest tolerance for manufacturing errors and for variation in the bottom turning point of driving corresponds to large $J_{12}$ and small $J_{21}$ while keeping $\gamma_{1}^{-}\left(+\right)$ below the smallest amplitude to be remembered. Crucially, hysteron pairs that fail to satisfy Eq. 2 do not corrupt an ensemble’s memory; they are simply absent from readout.

The scaling estimate presented above is a departure from analyses of *P* based solely on the ordering of thresholds, as considered by van Hecke (*21*): Here, we included the turning point of asymmetric driving, which additionally isolates the $J_{0}^{2}$ behavior by cutting out the zeroth-order response.

The two-hysteron latch is also how memories of asymmetric driving arise in larger groups of interacting hysterons, so that this motif may be observed in a bulk disordered material or metamaterial (*28*, *34*). Figure 5B shows nearly identical results for larger, mutually interacting groups after dividing out the multiplicity of frustrated pairs. These results hold even when we randomly make half of the interactions cooperative (drawn from $\left[0,J_{0}\right]$), strongly suggesting that Fig. 4D is the dominant mechanism despite many more possible behaviors (*19*–*22*). In table S1, we further show that the memory-forming portions of these larger groups tend to have the same kinematics and interaction strengths as in *N* = 2. This remarkable conservation is possible because hysterons that do not contribute to asymmetric readout are largely following return-point memory, and so their states and transitions vary little from cycle to cycle.

### Nesting memories of asymmetric driving

Last, we return to our original question: how a system with hysteresis may store multiple memories of asymmetric driving. We focus on the upper thresholds of the two-hysteron latch, $\gamma_{1}^{+}\left(-\right)$ and $\gamma_{2}^{+}\left(+\right)$, which we relabel $\gamma_{A}$ and $\gamma_{B}$ for convenience, with $\gamma_{B}>\gamma_{A}$. In Fig. 4D, and in general, these thresholds determine which state a latching pair lands in at the end of a shear cycle of amplitude $\gamma_{0}$. There are three possibilities: The latch may be left totally undisturbed in the − − state ($\gamma_{A}>\gamma_{0}$), it may be stuck in the + − state ($\gamma_{B}>\gamma_{0}>\gamma_{A}$), or it may be pushed all the way to + + so that it returns to the − − state by the end of the cycle ($\gamma_{0}>\gamma_{B}$).

#### Disordered systems

**Fig. 6.**
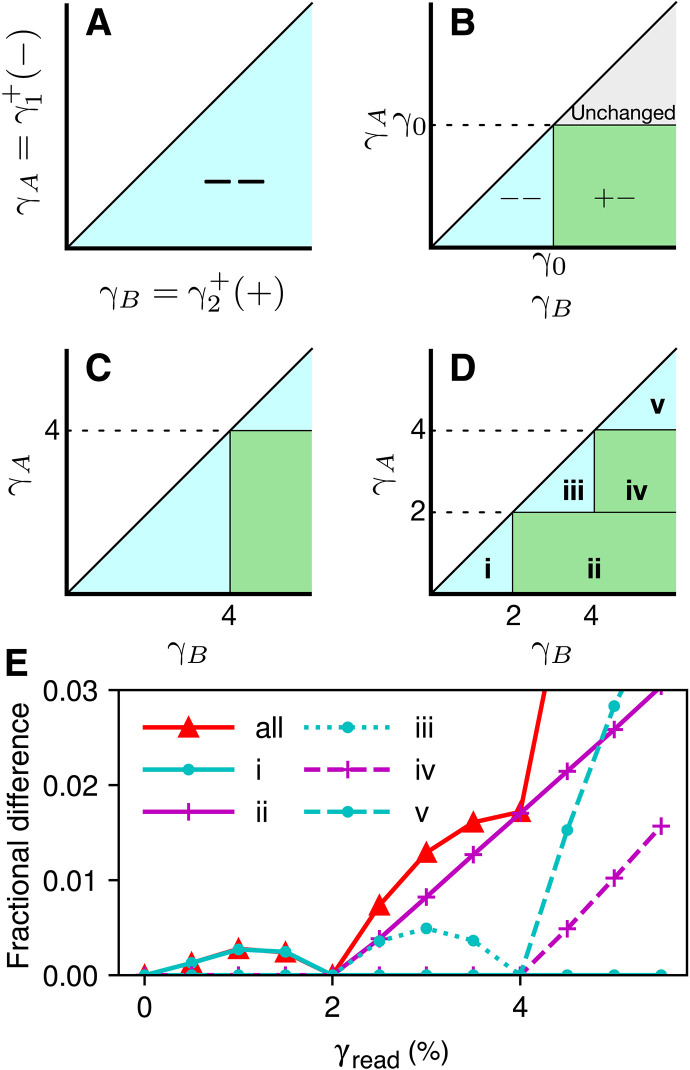
Graphical analysis of multiple memories. Each latching pair like Fig. 4D corresponds to a point on a plane, according to its upper thresholds. (**A**) An infinite ensemble of two-hysteron latches with continuously distributed thresholds, all initialized to the − − state. The thresholds are labeled $\gamma_{A}$, $\gamma_{B}$ for convenience. In accordance with Fig. 4D, $\gamma_{B}>\gamma_{A}>0$. (**B**) “Template” for how an asymmetric cycle with amplitude $\gamma_{0}$ changes the states of latches in the ensemble. (**C**) The ensemble in (A), after one cycle with amplitude 4% forms a memory. (**D**) The ensemble encodes two memories after cycles with 4 and 2% amplitude. (**E**) Readout of the ensemble in (D), reporting the fraction of hysterons in different states, in latches that conform to Fig. 4D and/or Fig. 5A. Curves show readout of all latches (“all”) and of ensembles generated separately to match each labeled region in (D).

An ensemble of many latches can be represented as a set of points on a γ_*B*_-γ_*A*_ plane, where γ_*B*_ > γ_*A*_ > 0, depicted in Fig. 6A with all latches initialized to − −. Figure 6B illustrates the result of a cycle with amplitude $\gamma_{0}$: Latches with $\gamma_{A}>\gamma_{0}$ are undisturbed, those with $\gamma_{B}<\gamma_{0}$ leave but return to the − − state, and all others go to the + − state. By applying the template of Fig. 6B repeatedly with different $\gamma_{0}$, we can graphically find the state of the ensemble after an arbitrary sequence of amplitudes. For example, in Fig. 6 (C and D), cycles of amplitude at 4% and then 2% write two memories, as in the “4, 2” protocol of Fig. 1B; the memories form a “stair-step” pattern on the plane.

To generate a readout signal like Fig. 4C, we apply cycles of increasing amplitude. The horizontal edge of the template, positioned at $\gamma_{0}$ in Fig. 6B, becomes a front that starts at zero and moves upward, changing pairs to + − as it passes; the vertical edge starts at zero and moves rightward, changing pairs to − −. In Fig. 6E, we show how each region marked in Fig. 6D contributes to a distinctive signal. Pairs in the closed regions “i” and “iii” begin readout as − −, are changed to + − by the horizontal front and add to the readout signal, and then are changed back to − − by the vertical front, forming non-monotonic contributions to the readout. For the pairs in regions “ii” and “iv” that were placed in the + − state by writing memories, the horizontal front has no effect, but the vertical front changes them to − −, so that their contribution to readout instead rises monotonically for all subsequent $\gamma_{\text{read}}$. Last, when readout surpasses the largest stored memory (4%), it reaches the triangular region “v,” which extends to the largest $\gamma_{A}$ and $\gamma_{B}$ allowed in our simulation, beyond the limits of the plots. This region’s contribution rises steeply as the horizontal front begins to sweep over it, falling only at much greater $\gamma_{\text{read}}$ (not plotted) when our finite ensemble becomes saturated. Together, the two stored memories create exactly two cusps in the combined readout signal, where its slope increases discontinuously.

The equivalence in Fig. 4 between a single hysteron under symmetric driving and a frustrated pair under asymmetric driving suggests that their multiple-memory capacities may be understood in the same way. Our method can describe the return-point memory of symmetric cycles that begin with positive strain, via the change of variables $\gamma_{A}\to -\gamma^{-}$, $\gamma_{B}\to \gamma^{+}$. However, our scheme is distinct from earlier graphical analyses of return-point memory for arbitrary driving (neither symmetric nor asymmetric) (*3*, *6*, *9*, *10*).

Unlike this idealized picture, in a disordered ensemble of physical hysterons such as an amorphous solid, the distributions of $\gamma_{A}$ and $\gamma_{B}$ obtained will be nonuniform and their range will be finite, according to the particular physics of the system. However, if these distributions are held constant, then the ability to encode more memories within a fixed strain interval, i.e., to form much finer stair steps than in Fig. 6D, is limited by the number of hysterons in the ensemble. In fig. S5, we extend our analysis to arbitrarily many memories, and we consider the lengths and areas of the stair steps of Fig. 6D to show that, as with return-point memory (*9*, *29*), the maximum number of nested memories scales with the square root of the ensemble size.

#### Designed systems and sequence recognition

The two-dimensional analysis in Fig. 6 can be made nearly one-dimensional if the hysterons’ parameters can be specified: Only some latches near the $\gamma_{A}=\gamma_{B}$ line are needed to encode the stair-step signature of nested memories, and the rest are redundant. We demonstrate this idea by constructing an ensemble. First, we divide the entire range of expected amplitude values into *M* nonoverlapping intervals: $\left[{\tilde{\gamma }}_{0},{\tilde{\gamma }}_{1}\right),\left[{\tilde{\gamma }}_{1},{\tilde{\gamma }}_{2}\right),\ldots ,\left[{\tilde{\gamma }}_{M-1},{\tilde{\gamma }}_{M}\right)$. The two endpoints of each interval then become $\gamma_{A}$ and $\gamma_{B}$ for each of *M* latches. As in the preceding discussions, finding the *m*th latch in the + − state is an evidence that a cycle with an amplitude between $\gamma_{A}={\tilde{\gamma }}_{m-1}$ and $\gamma_{B}={\tilde{\gamma }}_{m}$ was applied and that no cycle exceeding ${\tilde{\gamma }}_{m}$ has been applied since then. Thus *M* latches digitize and store *M* distinct amplitudes, a linear scaling, instead of the square-root scaling in a disordered ensemble. Together, the latches’ states are bits that distinguish one sequence of nested amplitudes from among $2^{M}$ possibilities. While a conventional single-dial combination lock does not rely on hysterons (instead using a series of wheels to store different values) (*1*, *2*), our analysis shows that it is possible to design interacting hysterons so that the functionality of a combination lock is obtained regardless of driving type.

## DISCUSSION

Return-point memory is a recipe for retaining arbitrarily many values from the history of a single variable, by coupling that driving to an ensemble of hysteretic elements. It has numerous examples in the natural world and in engineering, and, in many cases (when no frustrated interactions are present), it is the only possible behavior. Nonetheless, it fails whenever driving is asymmetric or rectified, as in a pedal depressed multiple times, or electrical signals from flashes of light.

Our results show that it is possible to store details of asymmetric driving if a system’s hysteretic elements interact. The behavior clearly violates return-point memory because the driving is bounded between two turning points, yet revisiting one of those points yields a new state. Nonetheless, the similarities with return-point memory are notable. Both mechanisms always store the most recent input but preserve past memories when amplitude is reduced, so that a system encodes the history of nested cycles of decreasing amplitude. Each kind of memory allows previous states to be recalled as amplitude is increased, yielding similar readout curves. Each arises from the smallest and simplest characteristic unit of its system: a “latch” formed by a single hysteron or an interacting pair. Last, each memory behavior is dominant for its respective driving type, even if these units interact with their environments, permitting the mechanisms to be highly scalable and defect-tolerant.

Together, these two behaviors point to a principle even more generic than return-point memory: robust, nested memories arising from units that latch at some input value and reset at a larger value, as in the parallel diagrams of Fig. 4 (B and D). For a single hysteron, this pattern is realized by the asymmetric placement of the flipping strains around 0, while, for the latching pair, one hysteron cannot return to a “down” state until a large deformation drives another hysteron “up,” the essence of a frustrated interaction.

Recent progress in creating and describing interacting mechanical hysterons (*13*, *14*, *23*, *34*, *35*) has now led to experiments with highly tunable interactions (*23*, *24*). Although the interactions in these experimental realizations may be more complex than in Eq. 1, the asymmetric latching behavior comes from a simple relationship among transition thresholds in an interacting pair (Eq. 2) and so lends itself to a variety of experimental systems, as was recently demonstrated with bistable rotors (*24*). We have shown that achieving nested memories and sequence recognition in these systems does not require one to fine-tune the competing interactions among multiple hysterons but instead comes from connecting independent latching pairs to a common drive.

Our work also suggests additional opportunities for the study of glassy matter. Even though frustrated interactions are essential to the physics of amorphous solids (*26*, *36*), crumpled sheets (*12*), and some magnetic systems (*18*, *32*, *37*) and mechanical metamaterials (*28*, *34*), in existing memory studies, frustration has largely been relegated to perturbing return-point memory. Our results show that a simple change to the driving protocol can suppress return-point memory and reveal a rich, intelligible, and distinctly glassy form of memory. Because this memory arises from a single dominant mechanism, even in larger systems, experiments and molecular dynamics simulations can characterize interaction strengths by tracking individual relaxations while varying the amplitude and origin of asymmetric driving. More generally, the readout method is based on differences, and so we look forward to results like those in Fig. 3B that quantify frustration in macroscopic samples via measurements of magnetization, light scattering, or even image subtraction (*9*).

Our study adds to the evidence that frustrated matter can remember what return-point memory must forget: that weakly breaking return-point memory tends to expand memory capacity. This hypothesis is also supported by studies of glassy systems’ ability to divide the frequency of driving (*19*–*22*) or retain vestiges of erased memories (*20*), although, in those examples, the mechanisms are unclear or lack a common motif, and they require *N* ≥ 3 hysterons. By contrast, the two-hysteron latch is a singular mechanism that is as small as possible yet scales linearly to store arbitrarily long sequences. The elementary principles and designs emerging from our work and from other recent studies hold promise for building mechanical information-processing systems as useful, robust, and ubiquitous as the venerable combination lock.

## References

[1] N. C. Keim, J. D. Paulsen, Z. Zeravcic, S. Sastry, S. R. Nagel, Memory formation in matter. Rev. Mod. Phys. 91, 035002 (2019).

[2] J. Junkunc, US Patent 948280 (1910).

[3] J. Barker, D. E. Schreiber, B. G. Huth, D. H. Everett, Magnetic hysteresis and minor loops: Models and experiments. Proc. Roy. Soc. A 386, 251–261 (1983).

[4] J. P. Sethna, K. Dahmen, S. Kartha, J. A. Krumhansl, B. W. Roberts, J. D. Shore, Hysteresis and hierarchies: Dynamics of disorder-driven first-order phase transformations. Phys. Rev. Lett. 70, 3347–3350 (1993). 10053845 10.1103/PhysRevLett.70.3347

[5] J. D. Paulsen, N. C. Keim, Mechanical memories in solids, from disorder to design. Ann. Rev. Cond. Mat. Phys. 16, 10.1146/annurev-conmatphys-032822-035544 (2024).

[6] N. C. Keim, J. Hass, B. Kroger, D. Wieker, Global memory from local hysteresis in an amorphous solid. Phys. Rev. Res. 2, 012004 (2020).

[7] M. Mungan, S. Sastry, K. Dahmen, I. Regev, Networks and hierarchies: How amorphous materials learn to remember. Phys. Rev. Lett. 123, 178002 (2019). 31702267 10.1103/PhysRevLett.123.178002

[8] C. W. Lindeman, S. R. Nagel, Minimal cyclic behavior in sheared amorphous solids. 10.48550/arXiv.2403.01679 [cond-mat.soft] (2024).

[9] N. C. Keim, D. Medina, Mechanical annealing and memories in a disordered solid. Sci. Adv. 8, eabo1614 (2022). 36197976 10.1126/sciadv.abo1614 PMC9534499

[10] F. Preisach, Über die magnetische Nachwirkung. Zeitschrift für Physik 94, 277–302 (1935).

[11] M. M. Terzi, M. Mungan, State transition graph of the Preisach model and the role of return-point memory. Phys. Rev. E 102, 012122 (2020). 32795063 10.1103/PhysRevE.102.012122

[12] D. Shohat, D. Hexner, Y. Lahini, Memory from coupled instabilities in unfolded crumpled sheets. Proc Natl Acad Sci USA 119, e2200028119 (2022). 35867743 10.1073/pnas.2200028119 PMC9282240

[13] H. Bense, M. van Hecke, Complex pathways and memory in compressed corrugated sheets. Proc Natl Acad Sci USA 118, e2111436118 (2021). 34876523 10.1073/pnas.2111436118 PMC8685682

[14] T. Jules, A. Reid, K. E. Daniels, M. Mungan, F. Lechenault, Delicate memory structure of origami switches. Phys. Rev. Res. 4, 013128 (2022).

[15] R. A. Guyer, “Hysteretic elastic systems: Geophysical materials” in *The Science of Hysteresis*, G. Bertotti, I. Mayergoyz, Eds. (Gulf Professional Publishing, 2006), vol. 3, pp. 555–688.

[16] I. D. Mayergoyz, Hysteresis models from the mathematical and control theory points of view. J. Appl. Phys. 57, 3803–3805 (1985).

[17] M. E. Semenov, S. V. Borzunov, P. A. Meleshenko, N. I. Sel’vesyuk, The Preisach model of hysteresis: Fundamentals and applications. Phys. Scr. 99, 062008 (2024).

[18] K. Binder, A. P. Young, Spin glasses: Experimental facts, theoretical concepts, and open questions. Rev. Mod. Phys. 58, 801–976 (1986).

[19] N. C. Keim, J. D. Paulsen, Multiperiodic orbits from interacting soft spots in cyclically sheared amorphous solids. Sci. Adv. 7, eabg7685 (2021). 34380623 10.1126/sciadv.abg7685 PMC8357233

[20] C. Lindeman, S. Nagel, Multiple memory formation in glassy landscapes. Sci. Adv. 7, eabg7133 (2021). 34380622 10.1126/sciadv.abg7133 PMC8357226

[21] M. van Hecke, Profusion of transition pathways for interacting hysterons. Phys. Rev. E 104, 054608 (2021). 34942848 10.1103/PhysRevE.104.054608

[22] A. Szulc, M. Mungan, I. Regev, Cooperative effects driving the multi-periodic dynamics of cyclically sheared amorphous solids. J. Chem. Phys. 156, 164506 (2022). 35490026 10.1063/5.0087164

[23] J. Liu, M. Teunisse, G. Korovin, I. Vermaire, L. Jin, H. Bense, M. van Hecke, Controlled pathways and sequential information processing in serially coupled mechanical hysterons. Proc Natl Acad Sci USA 121, e2308414121 (2024). 38768343 10.1073/pnas.2308414121 PMC11145188

[24] J. D. Paulsen, Mechanical hysterons with tunable interactions of general sign. 10.48550/arXiv.2409.07726 [cond-mat.soft] (2024). PMC13070039 41965852

[25] M. H. Teunisse, M. van Hecke, Transition graphs of interacting hysterons: Structure, design, organization and statistics. 10.48550/arXiv.2409.07726 [cond-mat.soft] (2024). PMC12457013 40994576

[26] A. Nicolas, E. E. Ferrero, K. Martens, J.-L. Barrat, Deformation and flow of amorphous solids: Insights from elastoplastic models. Rev. Mod. Phys. 90, 045006 (2018).

[27] K. Khirallah, B. Tyukodi, D. Vandembroucq, C. E. Maloney, Yielding in an integer automaton model for amorphous solids under cyclic shear. Phys. Rev. Lett. 126, 218005 (2021). 34114864 10.1103/PhysRevLett.126.218005

[28] C. Merrigan, C. Nisoli, Y. Shokef, Topologically protected steady cycles in an icelike mechanical metamaterial. Phys. Rev. Res. 3, 023174 (2021).

[29] I. Regev, I. Attia, K. Dahmen, S. Sastry, M. Mungan, Topology of the energy landscape of sheared amorphous solids and the irreversibility transition. Phys. Rev. E 103, 062614 (2021). 34271642 10.1103/PhysRevE.103.062614

[30] D. Fiocco, G. Foffi, S. Sastry, Encoding of memory in sheared amorphous solids. Phys. Rev. Lett. 112, 025702 (2014). 24484027 10.1103/PhysRevLett.112.025702

[31] M. Adhikari, S. Sastry, Memory formation in cyclically deformed amorphous solids and sphere assemblies. Eur. Phys. J. E 41, 105 (2018). 30206724 10.1140/epje/i2018-11717-5

[32] O. Hovorka, G. Friedman, Onset of reptations and critical hysteretic behavior in disordered systems. J. Magn. Magn. Mater. 290-291, 449–455 (2005).

[33] J. D. Paulsen, N. C. Keim, Minimal descriptions of cyclic memories. Proc. Roy. Soc. A 475, 20180874 (2019). 31293356 10.1098/rspa.2018.0874 PMC6598062

[34] C. Sirote-Katz, D. Shohat, C. Merrigan, Y. Lahini, C. Nisoli, Y. Shokef, Emergent disorder and mechanical memory in periodic metamaterials. Nat. Commun. 15, 4008 (2024). 38773062 10.1038/s41467-024-47780-w PMC11109184

[35] G. Muhaxheri, C. D. Santangelo, Bifurcations of inflating balloons and interacting hysterons. Phys. Rev. E 110, 024209 (2024). 39295065 10.1103/PhysRevE.110.024209

[36] D. Kumar, S. Patinet, C. E. Maloney, I. Regev, D. Vandembroucq, M. Mungan, Mapping out the glassy landscape of a mesoscopic elastoplastic model. J. Chem. Phys. 157, 174504 (2022). 36347694 10.1063/5.0102669

[37] I. Gilbert, G.-W. Chern, B. Fore, Y. Lao, S. Zhang, C. Nisoli, P. Schiffer, Direct visualization of memory effects in artificial spin ice. Phys. Rev. B 92, 104417 (2015).

