# Supplementary material for: Generalizing multiple memories from a single drive: The hysteron latch
Source: Sci Adv. 2025 Jan 29;11(5):eadr5933. doi: 10.1126/sciadv.adr5933 (PMC11778092; doi:10.1126/sciadv.adr5933)
Supplement: Supplementary file 1 — Supplementary Text Figs. S1 to S5 Table S1 Data S1 [file sciadv.adr5933_sm.pdf]

Science Advances
AAAS

Supplementary Materials for

Generalizing multiple memories from a single drive: The hysteron latch

Chloe W. Lindeman *et al.*

Corresponding author: Nathan C. Keim, keim@psu.edu

Sci. Adv. **11**, eadr5933 (2025)
DOI: 10.1126/sciadv.adr5933

This PDF file includes:

Supplementary Text
Figs. S1 to S5
Table S1
Data S1

Supplementary Text

Effect of initial condition

In the main text we consider asymmetric driving with positive strain only, and all hysterons beginning in the "–" state—opposite the polarity of driving, as shown in Fig. 1. Here we describe simulations that begin in the "+" state, as though $\gamma \to \infty$; in these simulations we drive the hysterons to $\gamma = 0$ and then write and read memories as in Figs. 1 and 2. Figure S1 shows these results along with the curves from Figs. 3 and 4(A, C). The effect on *symmetric* driving in Fig. S1(A, C) is subtle. However, the effect of initialization on return-point memory under *asymmetric* driving in Fig. S1(B, D) can be dramatic. In simulations without interactions ($J_{ij} = 0$) the asymmetric curve initialized with the same (positive) sign shows no memory at all—it has a fractional difference of 0 for all readout strains. This is a consequence of return-point memory: because γ is bounded between ∞ and 0, visiting the turning point at 0 after each cycle restores the system to its state before encoding. By contrast, the second-order memory mechanism illustrated in Fig. 4d is not return-point memory, and its behavior for $\gamma \leq 4\%$ is the same whether we first approach $\gamma = 0$ from $+\infty$ or $-\infty$. Thus in Fig. S1B, the two asymmetric protocols each form a memory, and their readout results agree for $\gamma_{\text{read}} < 4\%$. Differences at larger γ_{read} are due to hysterons that were not affected by writing and readout, and that retain only a memory of the initial condition.

Asymmetric driving with same-polarity initialization is thus a special case for return-point memory, and for simplicity we have omitted it from the main text. Other initialization protocols (*9*) will generally be able to form memories of single amplitudes under asymmetric driving, even in systems with perfect return-point memory. We note that the readout behavior past the stored memory (here, at $\gamma_{\text{read}} = 4\%$) is itself a kind of memory of initial conditions.

System parameters and the interaction strength scale

There are three strain scales that play a role in the effect of interactions on a pair of hysterons: the amount of hysteresis before adding interactions $\gamma^+ - \gamma^-$ (sometimes called the hysteron length), the interaction strength scale J_0, and the driving amplitude, taken here to be a few percent, $\gamma_0 \approx 0.04$. The effect of the driving amplitude is to select which hysterons are involved in the writing and readout process: only hysterons with hysteresis $\gamma^+ - \gamma^- \sim \gamma_0$ can contribute. Here, the hysteresis is

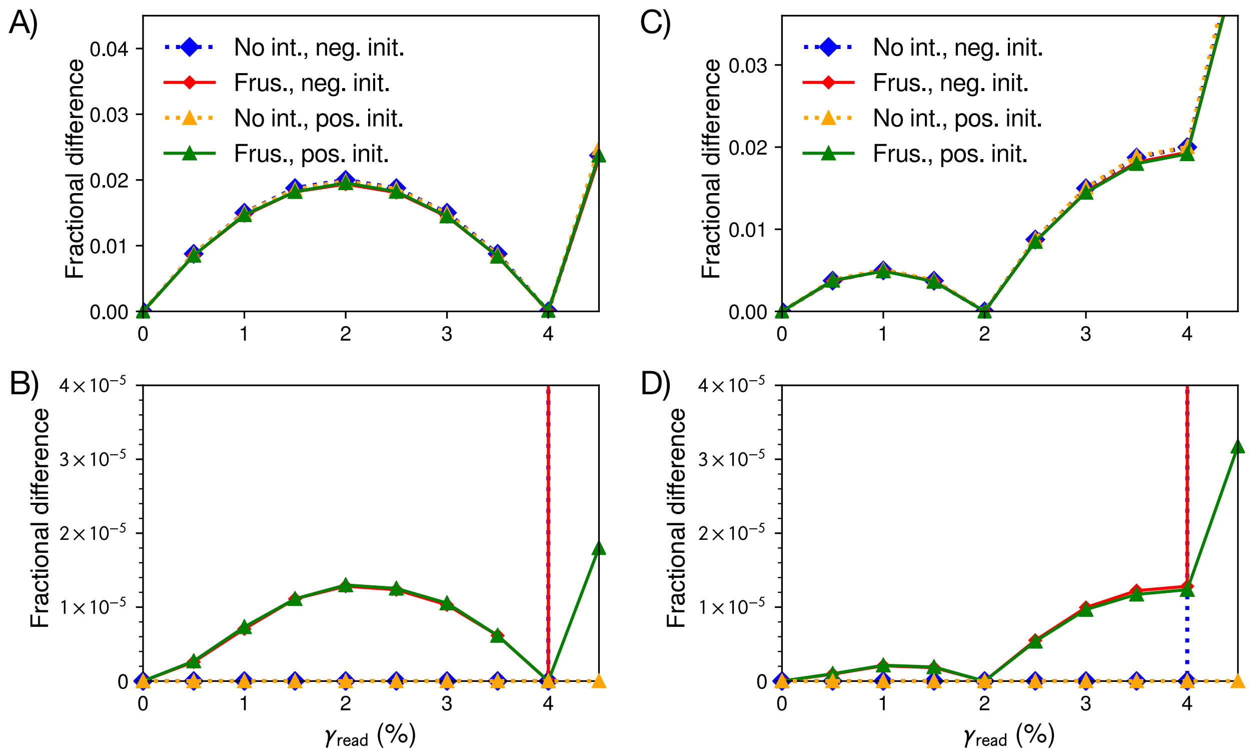

Figure S1: **Readout curves showing role of initialization for asymmetric driving.** Data are reproduced from Figs. 3 and 4(A,C), with the addition of frustrated ensembles starting from an all-positive initial state (i.e. the same polarity as driving). **(A)** Readouts after writing a single memory at $\gamma = 4\%$. **(B)** Readouts after writing memories at 4% and 2%. Positive initialization has no effect on the two-hysteron mechanism that gives rise to the signal for $\gamma_{\mathrm{read}} \leq 4\%$, while it changes the slope of the response at larger γ_{read}.

effectively chosen from $[0, 0.2]$, and the contributing hysterons are further cut down by $\gamma_0 \approx 0.04$. A natural non-dimensional scale of interaction strength would therefore be relative to the driving amplitude: $J^* \sim J_0/\gamma_0$. In our scaling study, J_0 ranges from 10^{-5} to 10^{-2}, giving J^* from $\sim 3\times10^{-4}$ to ~ 0.3.

Note that in other cases where hysteresis is small relative to the driving amplitude, the non-dimensional scale of interaction strength might be better defined as the ratio of J_0 to the hysteron length.

Readout curves for various J_0 and N

Figure S2 shows the readout curves, generated in the manner of Fig. 4c, that were used to measure memory signal strength for Fig. 4f. The "maximum memory signal" plotted in the Fig. 4f refers to the maximum value of each curve for $\gamma_{\text{read}} < 4\%$, and is measured only on curves that are non-monotonic, meaning that some larger values of J_0 are not plotted in the paper. We also omit measurements for $N = 2$, $J_0 < 10^{-4}$, for $N = 3$, $J_0 < 4.6 \times 10^{-5}$, and for $N = 9$, $J_0 < 2.2 \times 10^{-5}$ because in those cases the entire signal comes from at most 1 hysteron, making a measurement difficult to interpret.

Transition graphs and generalized inequalities

A particular group of hysterons can be represented as a transition graph, in which nodes are states of the hysterons (for example, ++ or +−) and arrows represent transitions between states for increasing and decreasing strain. For two hysterons, all such transition graphs are enumerated in (*21*). Of those, only three can contribute to the non-zero readout signal explored in Fig. 4. These transition graphs are shown in Fig. S3, with example values of the flipping strains given for each arrow in the cases observed in simulation. These example flipping strains satisfy Eq. 2.

Note that, in practice, the transition graph in Fig. S3B did not contribute substantially to the readout signals, as it requires large interaction strength relative to the length $\gamma_2^+ - \gamma_2^-$ of hysteron 2. We quantify this contribution in the bottom row of Table S1. The transition graph in Fig. S3C did not contribute at all because it requires breaking the rule used throughout the main text that each J_{ij}, J_{ji} pair must have the same sign (*21*).

To generalize the inequalities in Eq. 2 to include all three transition graphs, we allow for non-frustrated interactions. For the latching mechanism necessary for non-zero readout to occur, we still need one frustrated interaction (without loss of generality, $J_{12} < 0$), so the only new case allowed by this relaxation of strictly frustrated interactions is $J_{21} > 0$.

The general picture that emerges from the three contributing diagrams (shown in Fig. S3A–C) is (1) that the hysterons must travel through all four possible states in a cycle of sufficient size (rather than, for example, going through +− on both the way up and the way down); and (2) that the transition back from +− to −− is outside the range of driving, that is, below zero. This sets up

N	2	3	9	9± (a)	9± (b)
$P(\text{coop})$	0	0	0	1/2	1/2
J_0	1.00×10^{-2}	2.00×10^{-3}	2.00×10^{-3}	2.00×10^{-3}	1.00×10^{-2}
$P(\text{sig})$	2.07×10^{-5}	2.19×10^{-6}	8.71×10^{-6}	4.72×10^{-6}	7.82×10^{-5}
$P(\text{sig})/(N-1)(1-P(\text{coop}))J_0^2$	0.21	0.27	0.27	0.30	0.20
$P(\text{kine} \mid \text{sig})$	1	0.98	0.91	0.82	0.67
$P(\text{param} \mid \text{kine})$	1	0.24	7.21×10^{-2}	4.57×10^{-2}	4.69×10^{-2}
$P(\text{coop} \mid \text{kine})$				1.22×10^{-2}	6.09×10^{-2}
$\langle J_{12} \rangle / J_0$	−0.78	−0.80	−0.80	−0.79	−0.77
$\langle J_{21} \rangle / J_0$	−0.18	−0.20	−0.20	−0.21	−0.20
$P(\text{avalanche} \mid \text{kine})$	2.24×10^{-2}	0	0	0	2.20×10^{-2}

Table S1: **The pair mechanism of Fig. 4d dominates memory of asymmetric driving in larger groups of hysterons.** Analysis was performed on ensembles of 10^9 groups of size N (columns); for the two "9±" ensembles, pairs of J_{ij}, J_{ji} were made positive with probability $1/2$. Analysis identified hysterons that contributed to the readout signal ("sig"), that matched the kinematics of Figs. 4d and/or S3D ("kine"), and that had cooperative interactions ("coop") or horizontal avalanches as in Fig. S3D ("avalanche"). $P(\text{sig})$ is also rescaled according to Fig. 4f. Average interaction values $\langle J_{12} \rangle, \langle J_{21} \rangle$ are from hysteron pairs that match kinematics and have a frustrated interaction; subscripts refer to the corresponding hysteron indices in Fig. 4d. $\langle J_{12} \rangle, \langle J_{21} \rangle$ are remarkably consistent, suggesting that memory kinematics of a pair are driven primarily by their interaction, even in larger groups.

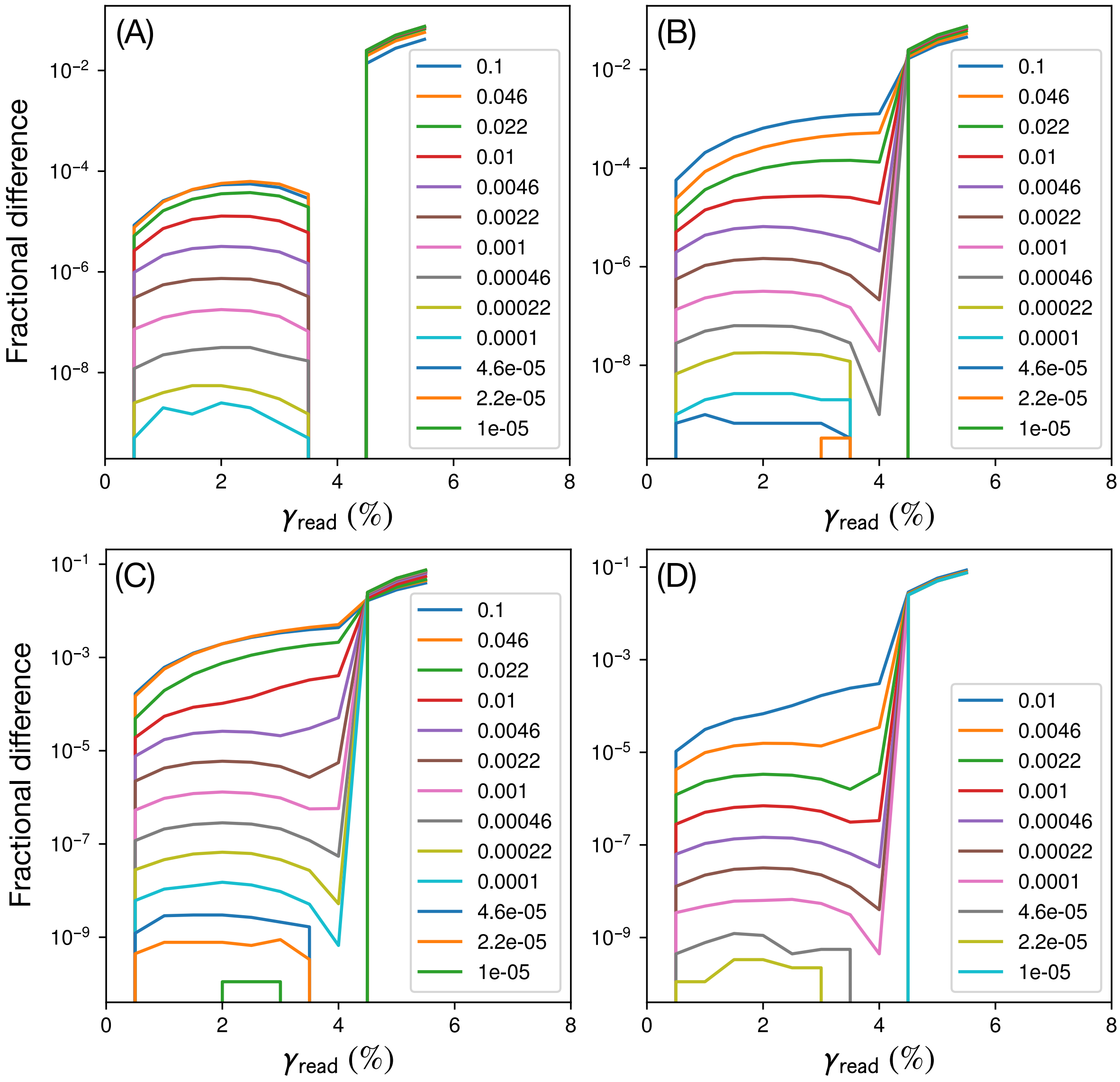

Figure S2: **Single-memory readout curves for various interaction strength scales J_0 and number of mutually-interacting hysterons N.** Each panel corresponds to a curve in Fig. 4f: (A) $N = 2$; (B) $N = 3$; (C) $N = 9$; (D) the "$N = 9\pm$" case where J_{ij}, J_{ji} pairs are made positive with probability 1/2. The legend identifies the value of J_0 for each curve and matches the vertical order of the curves; larger J_0 generally result in larger fractional differences. Fractional differences of zero are not shown on these logarithmic axes. Each curve was generated from an ensemble of 10^9 groups of N hysterons.

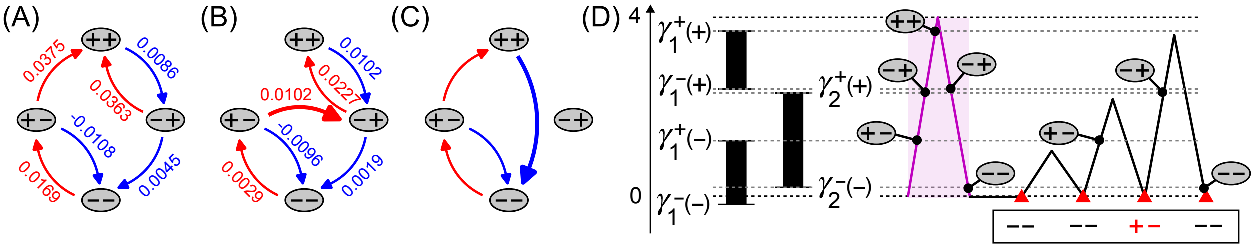

Figure S3: **Two-hysteron transition graphs that give rise to non-zero readout below the remembered strain.** Transitions encountered upon increasing (decreasing) strain are in red (blue). Examples of strain values that give rise to such a behavior are shown. (A) Transition graph corresponding to the example in Fig. 4d, also shown in Fig. 4e. (B) A second contributing transition graph, which occurs when $\gamma_1^-(+) > \gamma_2^+(+)$, also shown in Fig. 4e. This causes a so-called "horizontal" avalanche when the second hysteron flips to the + state under increasing strain (*21*). (C) A third contributing transition graph, which occurs when $\gamma_2^-(-) > \gamma_1^-(+)$. This causes a so-called "vertical" avalanche when the first hysteron flips to the − state under decreasing strain. No example strains are given because strictly negative interactions in the $N = 2$ pairs simulated prevented such a graph from contributing to readout. (D) Example hysterons in the strain-space format of Fig. 4(B,D) of the main text and corresponding to the type of transition graph shown in (B) (the flipping strains shown are not the exact values marked in (B); rearrangements have been spaced out for clarity).

the following inequalities:

- If we define hysteron 1 as the hysteron that flips forward first, then hysteron 1 should also be the first to flip back: $\gamma_1^-(+) > \gamma_2^-(+)$. This makes it possible to travel through all four states in a shear cycle.

- The transitions from ++ to −+ and from −+ to −− must occur at $\gamma > 0$. In other words, since we have already determined that hysteron 1 is the first to flip back to −, $\gamma_1^-(+) > 0$ and $\gamma_2^-(-) > 0$.

- The transition from +− to −− must be below zero: $\gamma_1^-(-) < 0$. This prevents the pair from always returning to the same state when the strain is returned to 0.

More concisely, the necessary and sufficient set of inequalities to get non-zero readout below the remembered strain for two interacting hysterons that have been driven asymmetrically is

$$\gamma_1^-(-) < 0 < \gamma_1^-(+) \tag{S1}$$

$$\gamma_2^-(+) < \gamma_1^-(+) \tag{S2}$$

$$0 < \gamma_2^-(-), \tag{S3}$$

which is satisfied by graphs S3(A–C) and in Figs. 4d and S3D, and of which Eq. 2 is the special case for $J_{21} < 0$.

Frustrated-Pair Mechanism in Larger Systems

The scaling analysis in Fig. 4f suggested that the memory of asymmetric driving in larger groups of N mutually-interacting hysterons (Fig. S2) was dominated by the action of frustrated pairs. While for $N = 2$ one can predict memory behavior by inspecting the model parameters (Eqs. 1 and 2), for $N > 2$ the numerous interactions and metastable states make this impractical. Instead, we can investigate the origins of memory readout signals in larger groups and ask whether the mechanism for memory in a pair is at work, by examining kinematics: the transitions of individual hysterons within larger groups, as a memory is written and read with the asymmetric protocol in Fig. 2c.

Table S1 summarizes our analysis of the kinematics in ensembles of 10^9 groups, generated according to the same schemes as the inset of Fig. 3b and Fig. S2. We fix the maximum interaction

strength at $J_0 = 2 \times 10^{-3}$, except $J_0 = 10^{-2}$ for $N = 2$, the N for which the memory behavior is least likely in a group. We first drive an ensemble with the single-memory asymmetric protocol in Fig. 2a. We identify the probability $P(\text{sig})$ that a given hysteron contributes to a non-monotonic memory readout signal, as in Fig. 4c — specifically, that its state after the cycle with $\gamma_{\text{read}} = 4\%$ matches its state at the beginning of readout, and that it is in a different state at the end of at least one intervening cycle.

Next, each hysteron that counts toward $P(\text{sig})$ belongs to several interacting pairs (for N hysterons, $N - 1$ pairs). For each pair, we extract the sequence of states that the pair visits in each cycle of readout. Figure 4b establishes that the memory behavior of interest arises from the distinct sequences obtained during small-, medium-, and large- amplitude cycles. To check whether these sequences match the progressions in Fig. 4d or Fig. S3D, our analysis implements the finite-state machine in Fig. S4; this also checks for a (rare) variant made possible by the avalanche behavior. We then ask: of the hysterons that contribute to the readout signal, what fraction $P(\text{kine} \mid \text{sig})$ belongs to exactly one pair that matches these kinematics? Remarkably, in Table S1 we report that $P(\text{kine} \mid \text{sig}) \sim 1$ even in large groups with cooperative and frustrated interactions ("9±").

We can further ask whether the actual mechanism of Fig. 4d, which is based on an isolated pair of hysterons, has any explanatory value for the pairs embedded in larger groups. At one extreme, one might hypothesize that these pairs are nearly isolated from their companions, so that their randomly-generated interaction *and* threshold parameters are often consistent with Eq. 2—a probability we measure as $P(\text{param} \mid \text{kine})$. Instead, Table S1 shows that $P(\text{param} \mid \text{kine}) \ll 1$ in large groups, meaning that the effects of other hysterons on the thresholds in Eq. 2 are significant; the behavior cannot be predicted from the two hysterons alone.

At the other extreme, one might hypothesize that a pair of hysterons with memory kinematics is merely a "puppet" of other hysterons with unknown memory behaviors, and that the interaction within the pair is so unimportant that it need not even be frustrated. In the "9±" ensembles where cooperative interactions occur with probability 0.5, we find that this "puppet" scenario is not supported, since $P(\text{coop} \mid \text{kine}) \ll 1$.

Most remarkably, Table S1 shows that for hysteron pairs that match kinematics and have a frustrated interaction, the mean interaction values $\langle J_{12} \rangle$, $\langle J_{21} \rangle$ are nearly the same fraction of J_0 in all ensembles. In other words, the kinematics of a memory-forming pair within a larger group are

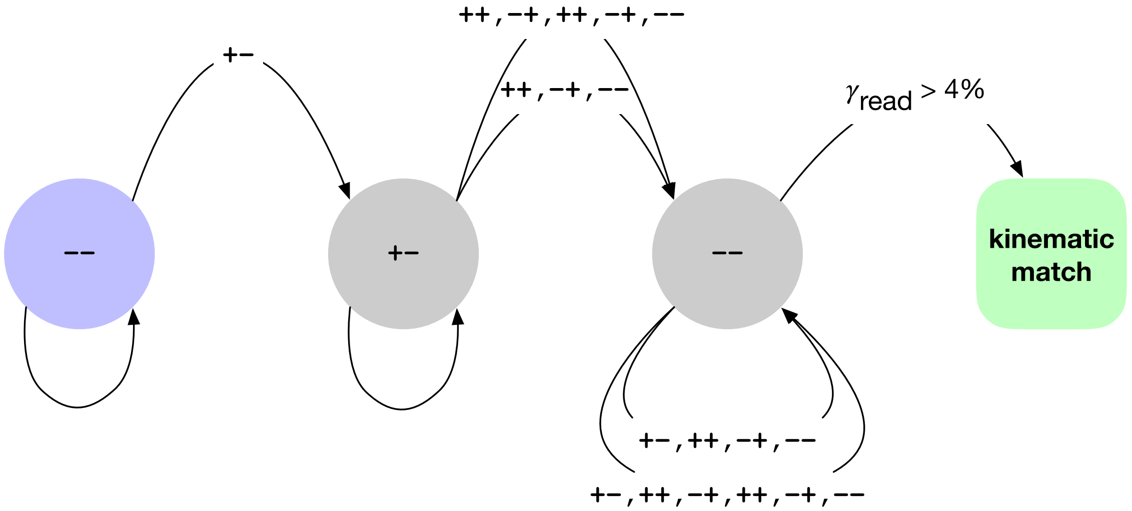

Figure S4: **Testing the kinematics of a hysteron pair.** A finite-state machine tests whether the kinematics of a memory-encoding pair during readout match the readout sequence shown in Fig. 4D or Fig. S3D. Each arrow represents one cycle of asymmetric driving and shows the sequence of states (if any) during that cycle; each node represents the state at the beginning/end of the cycle. Readout begins in the −− state at left and proceeds with increasing γ_{read}. To be accepted as a kinematics match, a hysteron pair must follow only an allowed sequence of states, and must be in the right-hand −− state after the cycle with $\gamma_{\text{read}} = 4\%$ (the strain amplitude that was written). The longer sequences from +− to −− and from −− to itself correspond to the uppermost transitions in Fig. S3B.

driven by the interactions within that pair, in the same way as for $N = 2$. Together, the results in Table S1 strongly support our interpretation of the J_0^2 scaling results in Fig. 4f: the mechanism in Fig. 4d is the dominant way that hysterons remember asymmetric driving, even in large systems with a mixture of cooperative and frustrated interactions.

Generalizing multiple-memory capacity in disordered ensembles

Here we generalize the template method outlined in Fig. 5 of the main text beyond the one- and two-amplitude writing sequences shown there. Figures S5(A–D) reproduce the initial case, the general template, and the one- and two-amplitude cases from Fig. 5(A–D). Figures S5(E, F) show the extension of these cases: it is possible to distinguish histories even when a larger number of amplitudes are written.

Following the argument of Keim and Medina (*9*), we consider briefly the memory capacity of a finite, disordered ensemble of frustrated pairs. Figure S5E adds a third memory, and illustrates that nested memories form a stair-step pattern on the plot. If the density of hysteron pairs on the plane is finite, then these stair-steps can be subdivided only so many times before they become indistinct. More precisely, if we generalize our memory values as the descending sequence $\gamma_n, \gamma_{n-1}, \ldots, \gamma_2, \gamma_1$, then memory $0 < m < n$ from within the sequence will be encoded by the pairs in the rectangle $\gamma_m < \gamma_B \leq \gamma_{m+1}$, $\gamma_{m-1} < \gamma_A \leq \gamma_m$. This can be seen by comparing Fig. S5E with the outcome if the 2% memory had not been written (Fig. S5F); the highlighted rectangle encodes this difference. If there is no pair of hysterons within such a rectangle, then the ensemble's state will be the same whether γ_m was written or not—the memory capacity has been exceeded. Thus, in a disordered ensemble of N latching pairs, the maximum number of distinct memories M scales as the maximum number of distinct rectangles that can be drawn in this way, such that each rectangle contains at least one pair. The linear spacing of these finely-drawn rectangles then scales as $1/M$, and the area of each one scales as $1/M^2$.

In the case of a disordered ensemble with roughly continuous distributions of γ_A and γ_B, the area of the smallest rectangle that contains one latching pair scales inversely with the number density of the pairs, leading to $1/M^2 \sim N$, for a memory capacity $M \sim \sqrt{N}$ as in the Preisach model (*9, 29*). The presence or absence of each memory corresponds to a different history, and so readout lets one distinguish among $\sim 2^M$ possible histories of asymmetric driving.

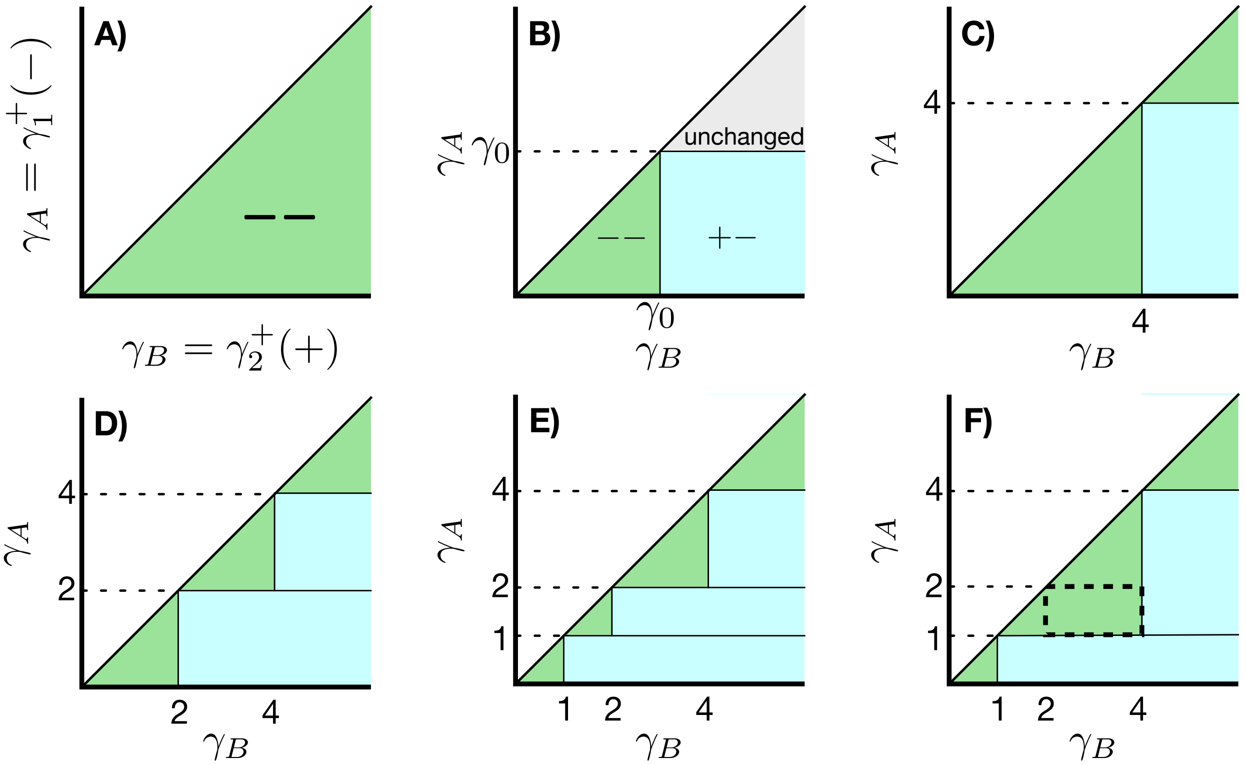

Figure S5: **Graphical analysis of multiple memories.** Each pair of hysterons like Fig. 4D can be represented as a point on a plane, according to its upper thresholds. (A) An infinite ensemble of such pairs with continuously-distributed thresholds, all initialized to the $--$ state. The thresholds are labeled γ_A, γ_B for convenience. In accordance with Fig. 4d, $0 < \gamma_A < \gamma_B$. (B) "Template" for how an asymmetric cycle with amplitude γ_0 changes the states of pairs in the ensemble. (V) The ensemble in (A), after one cycle with amplitude 4% forms a memory. (D) The ensemble encodes two memories after cycles with 4% and 2% amplitude. (E) Ensemble after cycles with 4%, 2%, and 1%. (F) Ensemble after cycles with 4% and 1%. Comparing with (E) reveals the set of pairs (outlined with heavy dashes) that encode the difference between these histories.

Caption for Data S1. Supporting data files. The data archive referenced in the main text, deposited on the Zenodo service and available at `https://doi.org/10.5281/zenodo.14002707`, contains a directory for each data plot and table in the text and Supplementary Materials. Each directory contains one or more CSV-formatted files containing the values for each curve in that plot. The directory names are listed below, along with notes where applicable:

- 2b-readout-symm-noninteracting — For clarity we avoided plotting curves that were identical (within random error), as is expected from the perfect return-point memory of these non-interacting hysteron systems. Thus, in both figures, the '2,4' curve was not plotted because it cannot differ significantly from the '4' curve. We provide the data files '24.csv' so that a reader can verify this agreement predicted by theory. In Fig. 2d, the '4,2' curve also matched the '4' curve, and so it was not plotted, but we provide the data file '42.csv' for verification.
- 2d-readout-asymm-noninteracting — See previous note.
- 3a-readout-symm-2mem
- 3b-readout-asymm-2mem
- 4a-readout-symm-1mem
- 4c-readout-asymm-1mem
- 4f-scaling — Data files are the un-rescaled maximum fractions different during readout; to rescale and obtain the values shown on the plot, see caption of Fig. 4f.
- 5e-readout-segmented
- S1a-readout-symm-1mem-polarity
- S1b-readout-asymm-1mem-polarity
- S1c-readout-symm-2mem-polarity
- S1d-readout-asymm-2mem-polarity
- S2a-int_strength_N=2

- S2b-int_strength_N=3
- S2c-int_strength_N=9
- S2d-int_strength_N=9pm
- table-S1-kinematics
